# JADPRO Live at APSHO 2015: Focus on Education and Collaboration

**DOI:** 10.6004/jadpro.2016.7.3.22

**Published:** 2016-04-01

**Authors:** Pamela Hallquist Viale

This past November, over 700 nurse practitioners, physician assistants, pharmacists, clinical nurse specialists, and other oncology professionals gathered at the JW Marriott Desert Ridge hotel in Phoenix to participate in the JADPRO Live at APSHO conference. This meeting was the third such JADPRO Live event focusing on the unique educational and professional needs of the advanced practitioner in hematology and oncology. 

The focus of the event in Phoenix was collaboration between all of the members of the oncology care team. Most of the presentations featured two speakers bringing different perspectives, educating the audience about not only the current state of management for a particular disease state, but also about collaborative practice and how it’s done at their institution: what works, what doesn’t, and what might work better in the future. 

**Figure 1:**
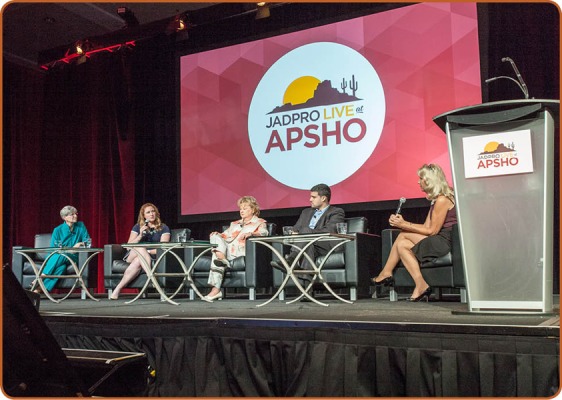


## THE CONFERENCE STORY

As conference chair and editor-in-chief of JADPRO, I’m very proud of the level of education that we’ve been able to bring to such a passionate group of advanced practitioners. Interestingly, the concept for the JADPRO Live conference grew out of the success of the print journal, the very first issue of which was published in 2010. Because we received so much great feedback about the value of the journal and the amount of education our readers were pulling out of the pages, we decided to "take the show on the road," as they say. 

Before long, we had partnered with long-time collaborator Meniscus Educational Institute (MEI) to accredit the content and therefore make the educational offerings even more valuable to JADPRO Live attendees.

At the conclusion of our first live meeting, which took place in St. Petersburg, Florida, in early 2014, APSHO was born: the Advanced Practitioner Society for Hematology and Oncology. At the next two live conferences in Orlando and Phoenix, the annual membership meetings of APSHO and its committees were held as well—hence expanding the name from JADPRO Live to JADPRO Live at APSHO.

## BACK TO THE JOURNAL

And now, in a way that I find somewhat poetic, we’ve come full circle: The live meeting content has come home to the print journal. In the pages of this very special issue of JADPRO, you’ll find summaries of all 19 of the main presentations that were given in Phoenix as well as the 2 lively panel discussions that took place.

Our staff writers have distilled each presentation into its key elements, with the hope of bringing you an overall sense of the meeting. I believe that when you digest the broad range of information presented in this special issue, you will be inspired to dig deeper into a particular subject. Or perhaps you’ll want to learn more about them all.

**Figure 2:**
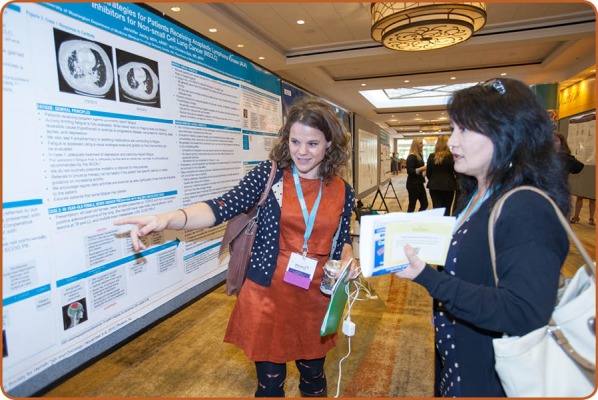


**Figure:**
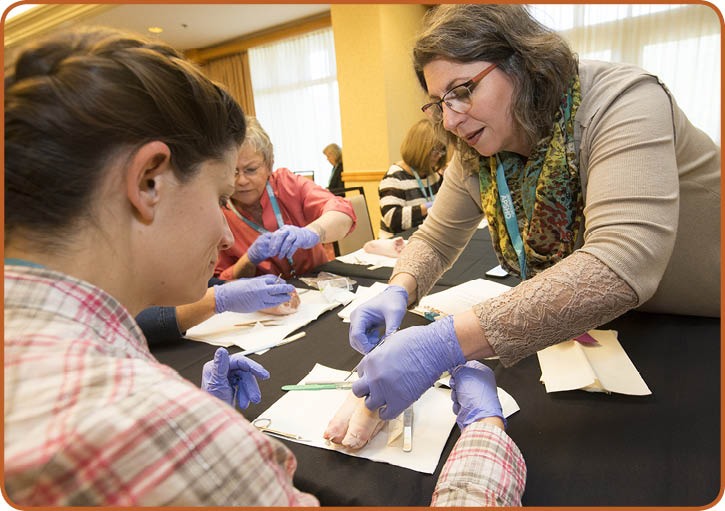


## TAKE THE EDUCATION FURTHER

If you do like what you see in these pages, you can take your education further by going to the JADPRO website (advancedpractitioner.com/sessions) to access 10 of the full presentations (with more to come in the upcoming month or so). There you’ll see all of the different learning formats that you can download: full-length video presentations, podcasts, slides, and written transcripts. Choose your preferred format, and enjoy the educational offering. Then, follow the instructions to complete a post-test and evaluation and earn your free CE/CME/CEU credits.

## SEE YOU IN DC: NOVEMBER 3–6, 2016

This special issue of JADPRO gives a good overview of the kind of educational content you’ll find at any of the JADPRO Live at APSHO conferences. But what you won’t necessarily see in these pages is the excitement and fun and passion that’s truly palpable as you walk around the conference site: in the main session rooms, the preconference workshops, the exhibit hall, the poster sessions, the luncheons and receptions…anywhere advanced practitioners gather and network with their peers. That energy comes from the feeling of being recognized, respected, and heard. And that’s what attendees tell us they feel at JADPRO Live. 

Please join us at the Gaylord National Hotel in National Harbor, Maryland (just outside of Washington, DC), from November 3 through 6, to participate in a truly unique educational experience. More information will be released soon, but in the meantime, visit jadprolive.com to see more about last year’s meeting. See you in DC!

—Pamela Hallquist Viale, RN, MS, CNS, ANP

Chair, JADPRO Live at APSHO

